# Yin-Dan-Ping-Gan Capsule Mitigates CCL_4_-Induced Liver Fibrosis via Regulating PPAR γ/GPX4 Signaling and Suppressing Ferroptosis

**DOI:** 10.3390/ph19020251

**Published:** 2026-02-01

**Authors:** Xue Jiang, Jicheng Yang, Yusheng Zhang, Ying Zhang, Zhen Ouyang, Chen Zhao, Limin Lin, Xianyu Li, Luqi Huang

**Affiliations:** 1 School of Food and Biological Engineering, Jiangsu University, Zhenjiang 212013, China; jianxue941213@outlook.com (X.J.);; 2 Beijing Key Laboratory of China Academy of Chinese Medical Sciences on Prevention and Treatment for Major Diseases, Experimental Research Center, China Academy of Chinese Medical Sciences, Beijing 100700, China; 3 Fujian Pien Tze Huang Enterprise Key Laboratory of Natural Medicine Research and Development, Zhangzhou Pien Tze Huang Pharmaceutical Co., Ltd., Zhangzhou 363000, China; 4 Institute of Chinese Materia Medica, China Academy of Chinese Medical Sciences, Beijing 100700, China

**Keywords:** Yindanpinggan Capsule, liver fibrosis, network pharmacology, proteomics, ferroptosis, PPAR γ

## Abstract

**Background:** Liver fibrosis is a major global public health issue that is only getting worse. The underlying molecular mechanisms of Yindanpinggan Capsule (YDPG), a traditional Chinese medication, are still unknown, although it has shown notable effectiveness in treating fibrosis and other forms of liver injury. **Methods:** To evaluate the impact of YDPG on liver fibrosis, a mouse model of liver damage caused by carbon tetrachloride (CCL_4_) was used. Proteomics, deep learning, network pharmacology, and later biological process validation using Western blot were used to elucidate the possible mechanism of YDPG in reducing liver damage. **Results:** Following YDPG treatment, we observed a decrease in the fibrosis index and an improvement in liver function. Network pharmacology, deep learning, and proteomics collectively identified the ferroptosis and peroxisome proliferator-activated receptor (PPAR) signaling pathways as pivotal in the anti-fibrosis effects of YDPG on the liver. Further experimental results showed that YDPG inhibited Malondialdehyde (MDA) and Fe^2+^ content and increased Glutathione (GSH) activity in fibrotic liver. Mechanistically, both SLC7A11/GSH pathway-mediated ferroptosis and oxidative stress up-regulated by the PPAR γ/GPx4 pathway were alleviated following YDPG treatment. **Conclusions:** Our present study corroborates that YDPG limits the progression of liver fibrosis by regulating the PPARγ-GPX4-ferroptosis pathway. These results indicate that YDPG could be a potential medication for hepatic fibrosis.

## 1. Introduction

Hepatic fibrosis is a chronic, fibro-proliferative disorder that can silently progress to cirrhosis or hepatocellular carcinoma (HCC) [1,2]. Viral hepatitis, metabolic-associated fatty liver disease, diabetes, iron overload, hereditary hemochromatosis, and chronic alcohol abuse are the main drivers that perpetuate hepatic injury [3]. In response to these insults, quiescent hepatic stellate cells (HSCs) become activated, secreting excessive extracellular matrix (ECM), pro-inflammatory cytokines, and proteases, thereby amplifying liver damage and fibrosis [3]. Without effective intervention, persistent fibrogenesis leads to end-stage liver failure or HCC, for which liver transplantation remains the only curative option [4,5]. Consequently, there is an urgent need to develop safe, anti-fibrotic therapies that can halt or reverse disease progression. To date, pharmacological strategies to block the transition from fibrosis to HCC have achieved limited success, largely because the molecular etiology of fibrotic liver disease remains incompletely understood [6]. Peroxisome proliferator-activated receptor gamma (PPAR γ), a ligand-activated nuclear hormone receptor, has emerged as a key metabolic regulator of hepatic lipid homeostasis and inflammation. Growing evidence implicates PPAR γ dysfunction in the pathogenesis of liver fibrosis and non-alcoholic fatty liver disease (NAFLD) [7]. Ferroptosis—an iron-dependent, regulated form of cell death driven by lethal lipid peroxidation—has also been recognised as a critical driver of hepatic injury; iron overload is a well-established risk factor for fibrosis progression [8]. Recent work demonstrates that PPARα can suppress iron-induced ferroptosis in mouse liver by up-regulating glutathione peroxidase 4 (GPX4) and transferrin receptor (TFR) [9], underscoring the therapeutic potential of targeting the PPAR–ferroptosis axis.

Chinese medicines have been used as multi-targeting agents to explore therapeutic approaches for liver fibrosis. Mori Fructus extract [10], E Se tea extract [11], and Gandankang aqueous extracts [12] have all shown anti-fibrotic activity in pre-clinical models. Chinese medicines are emerging as the most promising drug source for treating liver fibrosis. YDPG is a traditional Chinese Medicine (TCM) formula with properties of heat-clearing and dampness-removing, composed of *Artemisia capillaris* Thunb (Yin Chen)*, Gentiana scabra* Bunge (Long Dan), *Astragalus membranaceus* (Fisch) Bunge (Huang Qi), pig bile powder, *Gardenia jasminoides* J.Ellis (Zhi Zi), *Paeonia lactiflora* Pall (Bai Shao), *Angelica sinensis* (Oliv.) Diels (Dang Gui), and *Glycyrrhiza uralensis* Fisch (Gan Cao). Clinical studies have established its favorable safety profile and broad-spectrum hepatoprotective efficacy, notably mitigating acute alcohol-induced hepatic inflammation and oxidative stress. [13]. Still, the physiological mechanism of this response is not known.

To confirm the therapeutic impact of YDPG on liver injury, we created a mouse model of liver fibrosis caused by CCL_4_. The active ingredients and potential pharmacological mechanisms of YDPG in treating liver fibrosis were screened as targets by integrating network pharmacology and proteomics. Then, deep learning and Western blot were used to identify and validate YDPG targets further. Our findings provide a mechanistic framework for the development of YDPG as a multi-target therapy for hepatic fibrosis.

## 2. Results

### 2.1. YDPG Alleviates CCL_4_-Induced Liver Fibrosis

**Figure 1:**
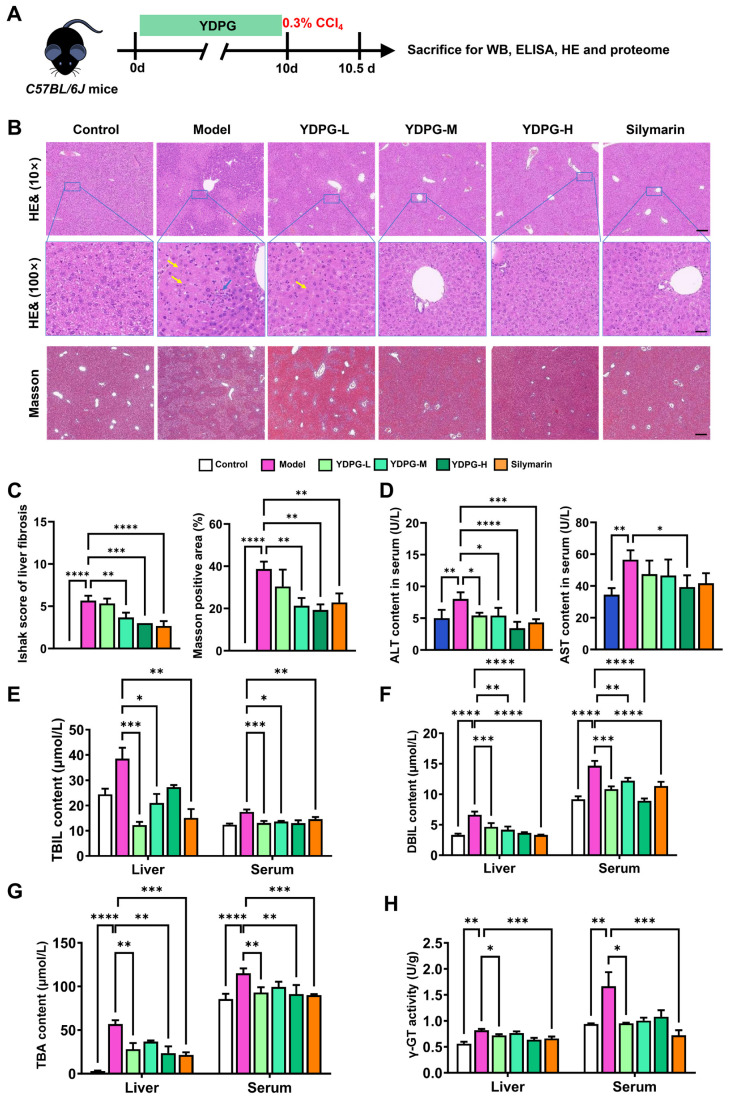
YDPG alleviates CCL_4_-induced liver fibrosis in mice. (**A**) Outline of experimental protocols for the CCl_4_-induced murine liver model. (**B**) Masson staining of liver slices, H&E, and representative liver images in the designated mice. Bar, 200 μm (10×) corresponds to 10 μm (100×). The sample size for each group is three. Coagulative necrosis in the hepatic lobule. The yellow arrow indicates hepatocyte edema; the blue arrow denotes infiltration of inflammatory cells. (**C**) Pathological scores and the positive area of Masson. (**D**–**G**) Activity of serum ALT and AST, along with serum and liver tissue concentrations of DBIL, TBIL, and TBA, in the designated animals. (**H**) γ-GT activity in the serum and liver of the specified animals. Values are presented as mean ± standard deviation (SD). One-way ANOVA accompanied by Tukey’s analysis was employed. * Denotes a statistically significant difference relative to the control group (**** *p* < 0.0001, *** *p* < 0.001, ** *p* < 0.01, and * *p* < 0.05).

We first evaluated the protective effect of YDPG against CCl_4_-induced liver fibrosis (experimental scheme in Figure 1A). At the end of treatment, biochemical and histological markers of liver injury were assessed. Compared with the model group, medium- and high-dose YDPG markedly reduced necrotic foci and inflammatory-cell infiltration (Figure 1B,C). Masson staining further revealed that CCl_4_ caused peri-portal and bridging fibrosis, whereas YDPG intervention significantly decreased collagen deposition, confirming its anti-fibrotic efficacy.

Serological analysis showed that all YDPG doses significantly lowered the elevated levels of Alanine aminotransferase (ALT), Aspartate transaminase (AST), Direct bilirubin (DBIL), and Total bilirubin (TBIL) induced by CCl_4_ (Figure 1D–F), while Total bile acid (TBA) remained unchanged (Figure 1G). Additionally, Gamma-glutamyl transferase (γ-GT) activity in both liver tissue and serum decreased dose-dependently with YDPG treatment (Figure 1H), indicating improved cholestasis and hepatocellular repair.

### 2.2. Analysis of Targets of YDPG for Improving Hepatic Fibrosis

**Figure 2:**
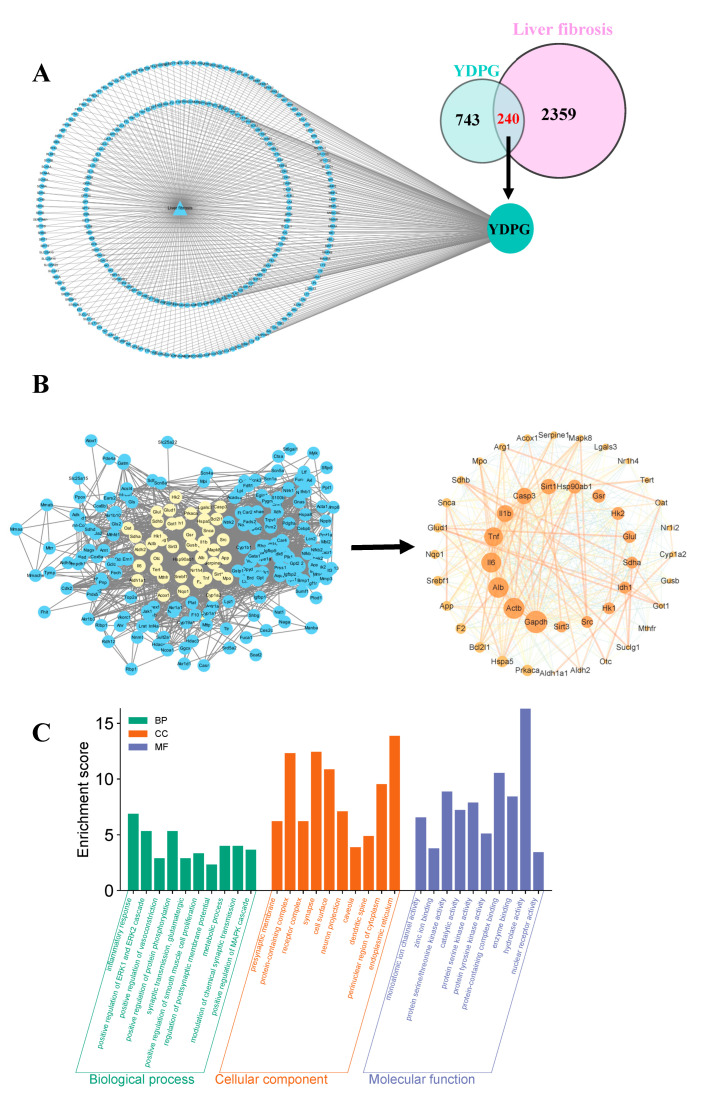
Analysis of targets of YDPG for improving hepatic fibrosis based on network pharmacology. (**A**) Target intersection VENN plot and common target maps for YDPG as well as liver fibrosis. (**B**) The key target pathway network diagrams of targets LF and YDPG capsule components. (**C**) Bar depicting the color gradient of Gene Ontology (GO) enrichment analysis, encompassing biological processes (BP), cellular components (CC), and molecular functions (MF), through Network Pharmacology.

Using network pharmacology analysis, we collected genetic data for liver fibrosis targets, identifying 2599 related genes, and mapped them against 983 active targets from YDPG capsule components, revealing 240 potential LF treatment targets via Venn analysis. The targets were examined in the String database and represented in Cytoscape 3.10.2 as a network including 241 nodes and 2091 edges, illustrating a polycentric interaction network that highlights the significance of core target proteins with a greater number of node connections (Figure 2A). We identified 45 core targets from the 275 components identified by UHPLC-Q-Orbitrap HRMS via Centiscape 2.2 and circularly ranked them based on network metrics (Figure 2B). The analysis of the GO function indicated their participation in several biological processes (BP), such as the response to inflammation, positive regulation of the MAPK cascade, and modulation of chemical synaptic transmission. Cellular components (CC) were predominantly localized in the presynaptic membrane, endoplasmic reticulum, receptor complex, and cell surface. Molecular functions (MF) encompassed monoatomic ion channel action, protein serine/threonine kinase activity, enzyme binding, and nuclear receptor activity. The signaling pathways with small P values and a substantial number of genes positively regulate the transcription of disease processes (Figure 2C).

### 2.3. Proteomics Revealed YDPG Broadly Reprograms Liver Proteins and Activates PPAR γ/GPX4 in CCl_4_-Treated Mice

**Figure 3:**
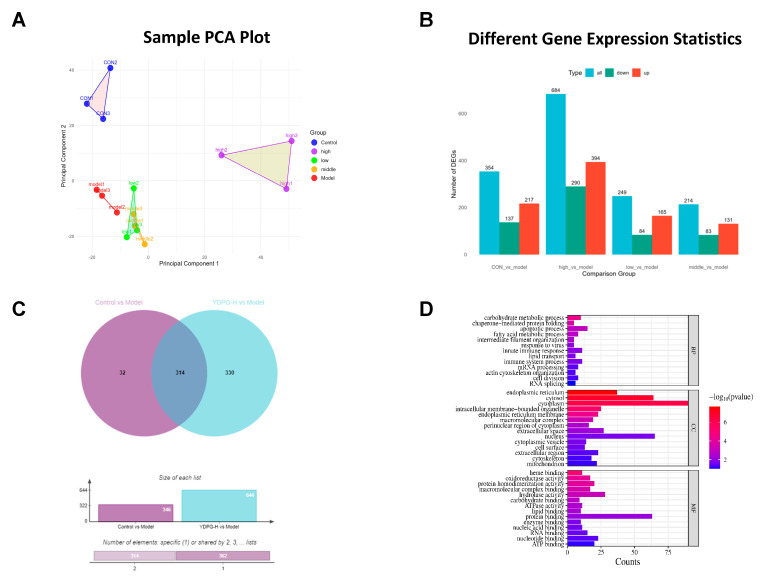
Proteomic changes in liver tissue of CCL_4_-treated mice after YDPG intervention. (**A**) Principal Component Analysis (PCA) of YDPG regulatory proteins. (**B**) The vertical bar of up-and down-regulated differentially expressed proteins (n = 3 denotes independent biological replicates). (**C**) Venn diagram of liver injury regulatory proteins and DYPG-H group regulatory proteins (n = 3). (**D**) Functional classification of proteins modulated or rescued by YDPG utilizing the Gene Ontology (GO) database.

Subsequently, we conducted a proteomic study of liver tissue in both control and model groups, identifying roughly 3496 proteins, of which 3230 were measurable. PCA clustering was conducted on the Control group, Model group, and YDPG group at varying concentrations, effectively distinguishing fibrotic liver, normal liver, and YDPG-treated liver by proteomics (Figure 3A). The measurable data were subjected to linear function normalization, thereby identifying differential proteins. Six hundred eighty-four differentially expressed proteins were identified by leveling these quantitative datasets via a linear adjustment. The statistical analysis of protein quantification differences between groups indicated that 217, 394, 365, and 131 proteins were up-regulated. In contrast, 137, 290, 84, and 83 proteins were down-regulated in comparisons of controls against model, and model versus high, low, and intermediate dose YDPG groups, respectively (Figure 3B). The Venn diagram shows 314 co-regulated differential proteins in the control group, model group, and high-dose administration group (Figure 3C). The 314 differentially expressed proteins underwent GO functional enrichment analysis from three perspectives (Figure 3D). The visual analysis of the top 14 activities indicates that differentially expressed proteins predominantly participate in biological processes (BP) including the apoptotic process, innate immune response, cell division, and fatty acid metabolic process, among others. From the standpoint of molecular functions (MF), differentially expressed proteins primarily execute regulatory roles within the cytoplasm, nucleus, endoplasmic reticulum, and other cellular compartments. The majority of the differentially expressed proteins originate from protein binding, hydrolase activity, and nucleotide binding (Cellular components, CC).

**Figure 4:**
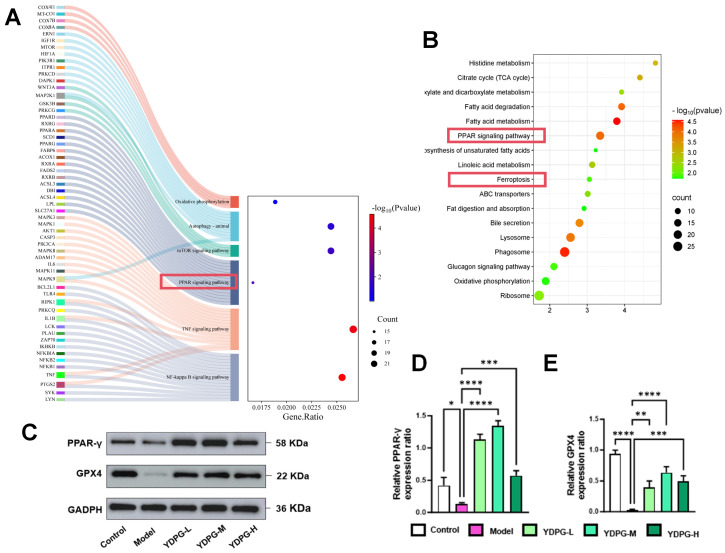
Effects of YDPG on PPAR γ/GPX4 pathway in CCL_4_-treated mice. (**A**) Target-pathway diagram of KEGG pathway analysis by Network Pharmacology. (**B**) Enrichment analysis of KEGG pathways by proteomics analysis. (**C**–**E**) Protein expression levels of PPAR γ and GPX4 were assessed (n = 3 denotes independent biological replicates). In comparison to the model group, statistical significance was observed with **** *p* < 0.0001, *** *p* < 0.001, ** *p* < 0.01, and * *p* < 0.05.

**Figure 5:**
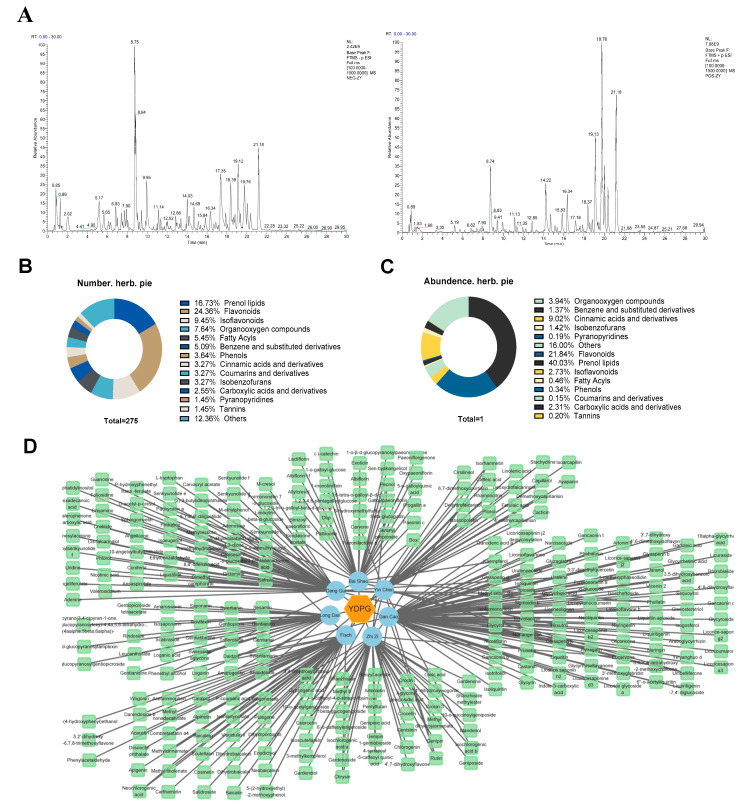
Component analysis of YDPG. (**A**) Base-peak ion flow diagram of YDPG in positive and negative modes. (**B**) The number of different types of compounds; (**C**) The relative peak area of different types of compounds. (**D**) Network analysis of herbs–compounds of YDPG.

Furthermore, functional enrichment analysis of these 684 proteins revealed that the differentially expressed proteins were primarily associated with the peroxisome proliferator-activated receptor (PPAR) signaling pathway and function, as well as several other signaling pathways. This is consistent with the predictions of network pharmacology (Figure 4A,B). KEGG pathway enrichment analysis indicated that the pathways included oxidative phosphorylation, the PPAR signaling pathway, ferroptosis (red box), and other signaling pathways (Figure 5B). Western blot analysis revealed a reduction in PPAR γ expression in the model group. Concurrently, it was markedly elevated in the YDPG group (Figure 4C,D). Additionally, we observed a decrease in GPX4 expression in the model group and an increase in the high-dose YDPG group (Figure 4C,E). These results suggest that YDPG attenuates hepatocyte injury induced by CCL_4_, which is associated with enhanced PPAR γ/GPX4 signaling pathway.

### 2.4. Identification of Chemical Constituents in YDPG

Samples were examined using UHPLC-Q-Orbitrap HRMS to identify the chemical constituents in YDPG. Figure 5A and Supplementary Table S4 present the base peak ion chromatograms and identification results of YDPG, respectively. In total, 275 chemical components were characterized: 76 in negative ion mode, 123 in positive ion mode, and 76 in both negative and positive ion modes. Details of the identified compounds mainly include 67 of Prenol lipids (16.73%), 46 of Flavonoids (24.36%), 26 of Isoflavonoids (9.45%), 21 of Organooxygen compounds (7.64%), 15 of Fatty Acyls (5.45%), 14 of Benzene and substituted derivatives (5.09%), 10 of Phenols (3.64%), 9 of Cinnamic acids and derivatives (3.27%), 9 of Coumarins and derivatives (3.27%), 9 of Isobenzofurans (3.27%), 7 of Carboxylic acids (2.55%), 5 of Pyranopyridines (1.45%) and Tannins (1.45%), and 104 of other compound (12.36%), in which relative peak area are 40.03%, 21.84%, 2.73%, 3.94%, 0.46%, 1.37%, 0.34%, 9.02%, 0.15%, 1.42%, 2.31%, 0.19%, 0.20 and 16.00%, respectively (Figure 5B,C). Specifically, *Glycyrrhiza uralensis* Fisch contributed 81 compounds, *Angelica sinensis* (Oliv.) Diels contributed 60 compounds, *Bunge* contributed 48 compounds, and *Paeonia lactiflora* Pall. contributed 40 compounds. In addition, *Artemisia capillaris* Thunb offered 28 unique components, while *Gentiana scabra* Bunge and *Gardenia jasminoides* J.Ellis contained 30 and 43 unique components, respectively (Figure 5D).

### 2.5. YDPG Prevents CCL_4_-Induced Hepatocyte Ferroptosis

**Figure 6:**
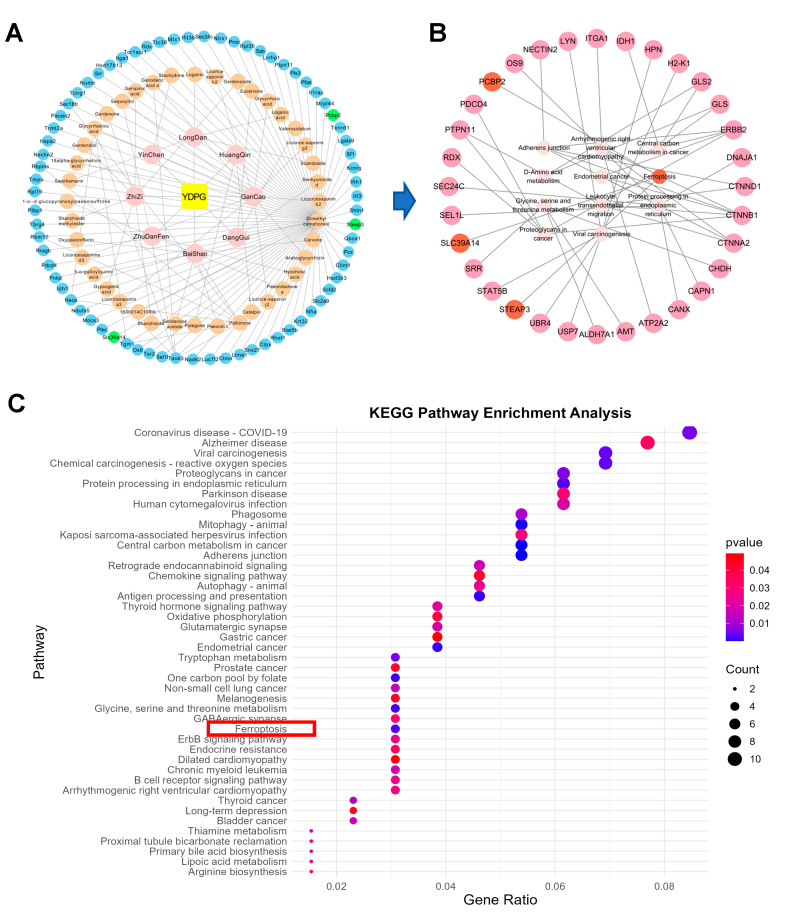
Deep learning predicts potential mechanisms of YDPG alleviating liver fibrosis. (**A**) Polycentric interaction network of drug-target interaction (DTI). (Pink: Herbals comprising YDPG; Orange: Compounds Blue: Targets; Green: Targets associated with ferroptosis). (**B**) Pathways associated with the primary target. (Light pink: pathway; Peach: target; Dark orange: targets associated with ferroptosis). (**C**) KEGG pathway analysis of 71 primary targets.

DeepPurpose is a molecular modeling and prediction toolkit utilizing deep learning to accurately forecast interactions between drugs and their targets. In this study, we utilized a drug-target interaction (DTI) prediction framework provided by DeepPurpose [14] to further reconfirm potential targets and pathways for YDPG treatment of CCl_4_-stimulated liver injury. A total of 314 common targets were identified through the comparison of differential proteins between the control group and the model group, as well as between the model group and the YDPG group. This reflects the multi-targeting property of TCM in disease treatment. Then DTI prediction of all relevant components of YDPG with significantly different proteins identified in control and model group proteomics studies yielded 59,096 docking results and their corresponding scores. Figure 6A illustrates that within the initial 200 docking results, YDPG for CCL_4_-induced liver injury comprises 40 active ingredients, including camphor dimethyl ester, palmitone, and glycyrrhizic acid, along with 71 target genes. These targets are primarily associated with ferroptosis, autophagy, the GABAergic ErbB signaling pathway, and the B-cell receptor signaling pathway (Figure 6B). KEGG pathway enrichment analysis indicated that YDPG targets are primarily associated with ferroptosis, autophagy, GABAe ErbB signaling pathway, and B cell receptor signaling pathway (Figure 6C). Notably, the DTI enrichment analysis predictions interestingly validated the Proteomics results involving ferroptosis (red box).

**Figure 7:**
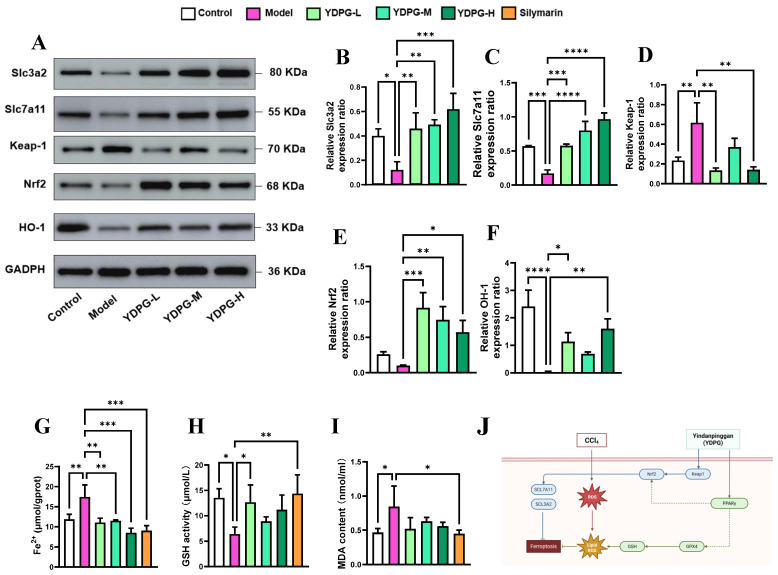
Effects of YDPG on hepatocyte ferroptosis in CCL_4_-treated mice. (**A**–**F**) The proteins expression of SLC3A2, SLC7A11, Keap-1, NRF2 and OH-1 (n = 3 denotes independent biological replicates). (**G**–**I**) The Fe^2+^, GSH, and MDA levels in serum and liver tissue. Compared to the model group, **** *p* < 0.0001, *** *p* < 0.001, ** *p* < 0.01, and * *p* < 0.05. (**J**) Schematic diagram for YDPG alleviating liver fibrosis by preventing hepatocyte ferroptosis via the PPAR γ/GPX4 pathway.

Research has shown that oxidative stress factors are a primary pathogenic mechanism contributing to the development of liver injury. Recent investigations have revealed that liver diseases, including liver fibrosis, are characterized by disturbances in iron metabolism and accumulation of lipid peroxides, indicative of ferroptosis [10,12]. Coincidentally, our proteomics results also show that the differentially expressed proteins of YDPG that regulate liver injury are involved in the ferroptosis pathway. We next examined the effects of YDPG on hepatocyte ferroptosis in CCL_4_-treated mice. The findings indicated an increase in the ferroptosis-related factors Fe^2+^ and MDA in the liver tissue of CCL_4_-treated mice, while YDPG decreased the levels of MDA and Fe^2+^ in the same tissue (Figure 7G–I). YDPG elevated the levels of ferroptosis inhibitory factors (SLC3A2, SLC7A11, NRF2, HO-1) in the liver tissue of CCL_4_-treated mice (Figure 7A–F). The PPAR γ/GPX4 axis may play a key role in YDPG-mediated improvement of hepatocyte ferroptosis (Figure 7J).

## 3. Discussion

Liver fibrosis is a deleterious clinical condition characterized by the excessive deposition of extracellular matrix proteins, which is prevalent in various chronic liver disorders [15]. The principal therapeutic approaches to impede liver fibrosis encompass liver vascular protection [16], suppression of hematopoietic stem cell activation, management of extracellular matrix development, and immunological modulation [16,17]. On one hand, research on nanofiber or mesoporous silica-based drug delivery systems facilitates the efficient delivery and controlled release of targeted drugs such as fluoxetine [18]. On the other hand, the development of anti-liver fibrosis drugs based on traditional Chinese medicine theory offers distinct advantages, including strong evidence-based support, multi-component formulations, multi-targeted approaches, and multi-pathway mechanisms. This study validated YDPG for the treatment of liver fibrosis. Through network pharmacology, deep learning, and proteomic analysis and validation with Western blot, YDPG may inhibit ferroptosis and oxidative stress to delay liver fibrosis progression in mice via the PPAR γ/NRF2/GPX4 axis.

CCl_4_ induces both acute and chronic hepatic damage, which is marked by hepatic lipid peroxidation, dysfunction, inflammation, fibrosis, and liver injury, all closely associated with cirrhosis and oxidative damage [19]. Recent studies reveal that the TGF-β/Smad axis plays a pivotal role in the fibrotic process [20]: this pathway not only directly initiates transcription of genes such as collagen and α-SMA, but also forms a positive feedback loop with oxidative stress—ROS activates Smad2/3, while Smad3/4 upregulates NOX4 and suppresses Nrf2, continuously amplifying oxidative damage. Previous studies have demonstrated that the natural product schisandrin B can block TGF-β/Smad signaling to alleviate CCl_4_-induced liver fibrosis in rats [21]. In mice, we observed that YDPG therapy reduced CCl_4_-induced liver damage and hepatic fibrosis, indicating that YDPG has a significant pathogenic effect on liver fibrosis. NF-κB, p53, PPAR γ, β-catenin/Wnt, and Nrf2 are among the transcription factors that can be activated by oxidative stress [22]. Our KEGG analysis showed that the targets screened by proteomics and network pharmacology were involved in the PPAR signaling pathway, while our Western blot data show that YDPG can increase PPAR γ expression. This signifies that the PPAR signaling pathway is essential in the mitigation of liver fibrosis by YDPG.

Ferroptosis is an iron-dependent, non-apoptotic cell-death modality that originates from severe redox imbalance and the bulk accumulation of phospholipid hydroperoxides [23]. Its core molecular determinants encompass (i) dysregulated lipid metabolism, (ii) depletion of the thiol buffer glutathione (GSH), and (iii) perturbed iron handling that increases the catalytically active Fe^2+^ pool [24]. Elevated free iron fuels the Fenton chemistry in which Fe^2+^ reduces H_2_O_2_ to hydroxyl radicals; these radicals abstract bis-allylic hydrogen atoms from polyunsaturated fatty acids (PUFAs), seeding lipid radicals that propagate peroxidation chains under O_2_ tension [25]. The resultant phospholipid hydroperoxides are normally detoxified via a GSH-dependent redox circuit [26]. Cystine import through the xCT antiporter (SLC7A11/SLC3A2) supplies the rate-limiting precursor for GSH biosynthesis [27], while glutathione peroxidase 4 (GPX4)—the only enzyme capable of reducing membrane lipid hydroperoxides—consumes GSH to abort the peroxidation cascade [28]. GPX4, the sole enzyme capable of reducing membrane lipid hydroperoxides, is transcriptionally and enzymatically disabled by the piR-16404/CASTOR1/mTORC1 axis upon toxic challenge with DMF: down-regulation of piR-16404 de-represses CASTOR1, blunts mTORC1 signaling, and consequently attenuates GPX4 synthesis, while parallel GSH depletion inactivates residual GPX4 [29]. The resulting unrestrained lipid ROS accumulation precipitates ferroptotic plasma-membrane rupture, leakage of hepatic enzymes (ALT/AST), and acute liver injury. This indicates that hepatic ferroptosis is a key factor in the pathophysiological mechanisms and progression of liver fibrosis, and inhibiting hepatic ferroptosis represents a potential therapeutic approach for alleviating liver fibrosis. This was also interestingly validated by our proteomics and DTI enrichment analysis results. Therefore, we further verified by Western blotting that YDPG significantly reduced CCl_4_-induced Fe^2+^ accumulation, increased the lipid peroxidation end product MDA, and decreased GSH. In YDPG-treated livers, increases in SLC7A11 and SLC3A2 associated with GSH synthesis were also detected. This suggests that YDPG alleviates the liver injury caused by CCl_4_ by rescuing the ferroptosis tendency.

Through an integrative analysis combining network pharmacology, deep learning, and proteomics, we have identified that both ferroptosis and the PPAR signaling pathway play crucial roles in the antifibrotic effects of YDPG on the liver. However, whether these two pathways are interconnected or interact with each other remains an open question. Earlier reports showed that ligand-activated PPAR γ can transcriptionally re-activate NRF2—a master regulator of lipid-peroxidation and iron metabolism [30]—and its canonical target GPX4 [31], thereby enhancing antioxidant defenses and inhibiting ferroptosis [32]. Our Western blot results indicate that YDPG attenuates the ferroptosis induced by CCl_4_, while simultaneously increasing the protein expression of NRF2. We detected changes in NRF2 and its downstream target OH-1, which is central to the regulation of the antioxidant molecule [25] and plays a key role in attenuating lipid peroxidation and iron metabolism [33]. In addition, KEAP1, a negative regulator of NRF2, was also downregulated by YDPG [34]. Therefore, we speculate that YDPG may prevent CCl_4_-induced liver fibrosis by activating PPAR γ. Activation of PPAR γ restores the expression of GPX4 and NRF2, enhances antioxidant defenses, and inhibits ferroptosis. These findings establish the PPAR γ/GPX4 axis as a crucial regulatory pathway in CCl_4_-induced liver fibrosis and identify YDPG as a promising therapeutic candidate for targeting ferroptosis during liver fibrosis.

In conclusion, we demonstrated that YDPG mitigated CCl_4_-induced liver injury by restoring function and structure, effectively delaying the progression of liver damage in mice. We then identified the major chemical constituents of YDPG via UHPLC-Q-Orbitrap HRMS, screened the top 45 compounds, and predicted key pathways associated with YDPG therapy using network pharmacology screening for hepatic fibrosis targets. Significantly different proteins were further identified by integrating deep learning-based drug target prediction results with proteomics data. KEGG pathway analysis of the aggregated results revealed that the selected targets were linked to ferroptosis and PPAR γ/GPX4 signaling pathways. This study presents a potential therapeutic approach for treating liver fibrosis.

Nevertheless, our study has only examined a limited number of pathways and associated targets. Whether the PPAR γ/GPX4 axis constitutes a key regulatory pathway for YDPG in alleviating hepatic ferroptosis requires further experimental validation. Additional targets along this pathway must be verified to comprehensively elucidate the mechanism of action of YDPG in liver fibrosis.

## 4. Materials and Methods

### 4.1. Chemical and Material

Zhangzhou Pien Tze Huang Pharmaceutical Co., Ltd. (Zhangzhou, China) supplied the YDPG. Mackin (Catalog No. C805325, Shanghai, China) supplied carbon tetrachloride (CCL_4_). Tianjin Tasly Pride Pharmaceutical Co., Ltd. supplied Silymarin (Cat. No. S2092, Tianjin, China).

### 4.2. Animals

Eight-week-old male C57BL/6 mice were acquired from Speyford Experimental Animal Science and Technology in Beijing, China (No.100332241100053036), and permitted to acclimate for one week before experimentation. During the trial, the animals were maintained in a controlled environment featuring a 12 h light/dark cycle, a temperature of 23 ± 1 °C, humidity levels between 50% and 60%, and unrestricted access to water and pelletized chow. All experimental procedures adhered to the Animal Care and Use Guidelines of the China Academy of Chinese Medical Sciences and the Principles of Laboratory Animal Care (protocol code ERCCACMS11-2209-04, approved on 26 September 2022).

### 4.3. Experimental Design

A total of 60 mice were allocated into 6 groups: model group, positive drug group, low-dose YDPG group, middle-dose YDPG group, high-dose YDPG group, and control group. Over ten days, both experimental and control groups received 0.2 mL of distilled water. Mice in the positive drug group were administered Silymarin at a dosage of 150 mg/kg. In contrast, the low-dose, middle-dose, and high-dose YDPG groups received 200 mg/kg, 400 mg/kg, and 800 mg/kg of YDPG, respectively, orally once daily for a duration of 10 days. On day 10, mice in the control group were given an intraperitoneal injection of olive oil (10 µL/g), whereas mice in the other four groups received olive oil (10 µL/g) with 0.3% CCl_4_. All mice were euthanized 12 h after the final dose of CCl_4_ (or olive oil) to facilitate the collection of blood and tissue samples [35]. Plasma was collected and centrifuged at 3500 rpm for 10 min at 4 °C to isolate the supernatant (serum), which was thereafter kept at −80 °C. Until further examination, the livers were either fixed in 4% or 10% buffered paraformaldehyde or frozen and maintained in liquid nitrogen. Both YDPG and silymarin were suspended in 0.5% CMC-Na.

### 4.4. Evaluation of Blood and Liver Parameters

Mouse liver function was assessed by measuring serum levels of alanine aminotransferase (ALT), aspartate transaminase (AST), direct bilirubin (DBIL), total bilirubin (TBIL), gamma-glutamyl transferase (γ-GT), and total bile acid (TBA). These measurements were conducted by Wuhan Servicebio Technology Co., Ltd. (Wuhan, China) utilizing a Chemray 240 automatic biochemical analyzer (Rayto, Shenzen, China). The liver tissues were analyzed for DBIL (Cat. NO. E-BC-K761-M), TBIL (Cat. NO. E-BC-K760-M), γ-GT (Cat. NO. E-BC-K126-M), and TBA (Cat. NO. E-BC-K181-M) using commercial kits provided by the Nanjing Jiancheng Bioengineering Institute in China (Nanjing, China), as per the manufacturer’s instructions.

### 4.5. Histological Analysis

Liver samples were fixed in a 4% formaldehyde solution at room temperature for 48 h, dehydrated in ethanol, cleared in xylene, embedded in paraffin, and sectioned into 5 μm slices for pathological examination using standard H&E staining (Wuhan Servicebio Technology Co., Ltd., Wuhan, China). Liver fibrosis was assessed through Masson’s trichrome staining (Wuhan Servicebio Technology Co., Ltd., Wuhan, China). Liver fibrosis and inflammation in H&E-stained sections were assessed using the Ishak index score (Tables S1 and S2), a commonly employed grading system for liver fibrotic disease [36]. Quantification was conducted using ImageJ (version 2.15.1, https://fiji.sc), developed by the National Institutes of Health, USA, was utilized to quantify both the area of individual egg granulomas and the total granuloma area. The quantification of positively stained regions in Masson red staining slices was conducted using ImageJ software.

### 4.6. Network Pharmacology Research

#### 4.6.1. Prediction of YDPG Component Targets

The chemical components of YDPG were investigated using a hybrid quadrupole orbitrap high-resolution mass spectrometer combined with ultra-performance liquid chromatography (UHPLC-Q-Orbitrap HRMS). Mass spectrometry data were analyzed using Progenesis QI 3.0 (Waters Corp., Milford, MA, USA). The identification was established through an analysis of the reference substance database (TCM Pro 2.0, Beijing Hexin Technology Co., Ltd., Beijing, China) and a theoretical database compiled from public databases and literature. The assessment focused on parameters including peak area, isotope distribution, retention time error, mass error of the parent ion, and congruence of daughter ions. The molecular structures of these components were represented using SMILES codes sourced from the PubChem database. The standardized SMILES codes were subsequently uploaded to the SwissTargetPrediction platform for potential target identification.

#### 4.6.2. Prediction of Liver Fibrosis Targets

To predict the targets for liver fibrosis (LF), we conducted searches using keywords such as “liver fibrosis.” Databases including GeneCards (https://www.genecards.org/, accessed on 1 July 2024), DisGeNet (https://www.disgenet.org/, accessed on 1 July 2024), and OMIM (https://omim.org/, accessed on 1 July 2024) were utilized for target discovery. The identified targets were standardized using the UniProt database, and the accuracy of their official gene names was verified. The standardized targets of the active components from step 2.3 and the LF disease targets were uploaded to a bioinformatics plotting platform (http://www.bioinformatics.com.cn/, accessed on 1 July 2024) to identify potential targets of action for YDPG capsules in the treatment of LF.

#### 4.6.3. Construction of Protein–Protein Interaction (PPI) Network

YDPG capsules’ possible therapeutic targets for LF were entered into the String database. “Mus musculus” was chosen as the species, and the lowest interaction score threshold was set at the highest level of confidence (0.9). Proteins not connected to the network were hidden, while all other settings were maintained at their default values. The resulting PPI data were saved in TSV format under the Exports section and then imported into Cytoscape software. The network was analyzed using the Centiscape 2.2 plugin to determine the degree and betweenness centrality (BC) values of each target. The network diagram was then visualized, with the size and color intensity of each node representing the magnitude of the values, aiding in the identification of key targets.

#### 4.6.4. Construction of Target-Pathway Network Diagrams

Cytoscape 3.7.2 was used to create the “key target pathway network” diagrams, and the Network Analyzer plugin was used to examine the network topology.

#### 4.6.5. Kyoto Encyclopedia of Genes and Genomes (KEGG) Pathway Enrichment Analysis and Gene Ontology (GO) Function

The potential targets of action were introduced into the DAVID database, with the gene species specified as “Mus musculus.” GO function and KEGG pathway enrichment analyses were conducted on these targets to explore the biological processes and associated signaling pathways involved in the therapeutic effect of YDPG capsules on LF.

### 4.7. Drug Target Interaction Prediction

Forecast all pertinent fractions of YDPG for proteins discovered in proteomics studies of the control and model groups, exhibiting substantial changes, utilizing the Drug-Target Interaction (DTI) prediction framework offered by DeepPurpose: the Graph-CNN-CPI architecture was selected as the core model because it couples a graph convolutional neural network operating on SMILES-derived molecular graphs with a CNN encoder that processes full-length protein sequences, an ensemble that consistently achieves state-of-the-art performance on the KIBA benchmark. Hyper-parameter configuration was as follows: learning rate 1 × 10^−3^, batch size 256, maximum training epochs 100, and early-stopping patience of 5 epochs to mitigate over-fitting. To ensure stringent biological relevance, we imposed a high-confidence cut-off: only compound–protein pairs exhibiting a predicted binding probability ≥ 0.90 (top 5% of all scores) were retained for downstream analysis. The complete YDPG compound library and the curated fibrosis-related protein set were randomly split into 80% training, 10% validation, and 10% test subsets using DeepPurpose’s built-in data_process function; the model was trained end-to-end on the training set, monitored on the validation set, and final predictions were generated from the held-out test set. This rigorous pipeline yielded a concise list of YDPG constituents with the highest likelihood of engaging the proteomics-identified targets, thereby prioritising molecules most plausibly responsible for the observed anti-fibrotic activity. All datasets, models, documentation, installation guidelines, and tutorials may be accessed at https://github.com/kexinhuang12345/DeepPurpose, accessed on 1 July 2024.

### 4.8. Proteome Analysis

The sample preparation procedure encompassed protein denaturation, reduction, alkylation, tryptic digestion, and peptide purification. Initially, 200 μL of 8 M urea solution and 0.1 M Tris/HCl at pH 8.5 (designated as UA buffer) were introduced to the sample, followed by mixing at 4000 rpm for 15 min at 4 °C post-grinding. The pH was subsequently modified by the addition of 100 mM NH_4_HCO_3_. Subsequently, 0.05 M iodoacetamide was introduced to the filter and incubated for 20 min in the absence of light. Subsequently, Trypsin (Promega, Madison, WI, USA) digestion buffer was introduced, and the mixture was incubated for 2 h at 37 °C with shaking at 500 rpm, followed by an overnight incubation at 4 °C. Introduce a formic acid solution to achieve a final concentration of 1%. The sample underwent centrifugation at 12,000× *g* for 5 min, after which the supernatant was subjected to further centrifugation in pre-activated SEP-PAK VAC C18 cartridges (Waters, USA) using 0.1% formic acid (FA). The method led to the adsorption of peptides derived from enzymolysis onto the C18 cartridges. The C18 cartridges were rinsed with 50% acetonitrile (CAN) and 0.1% TFA, resulting in the acquisition of the desorbed pure peptide solution. The isolated peptide segments were lyophilized and kept at −20 °C. The samples underwent DIA data acquisition using an Acquity M-class high-performance liquid chromatography (HPLC) system (Waters) in combination with a ZenoTOF 7600 mass spectrometer (Sciex, Concord, ON, Canada) equipped with an OptiFlow Turbo V ion source and run by SciexOS 3.1 software (AB Sciex version 2015) before analysis. The samples were reconstituted with an injection buffer. In the end, data analysis was conducted using the DIA-NN program (version 1.8.1) to get both qualitative and quantitative protein results.

### 4.9. Antioxidant Enzyme Activities and Fe^2+^ Levels Detection

Malondialdehyde (MDA) levels were quantified using a malondialdehyde test kit via the thiobarbituric acid reactive substance method (Cat. NO. G4302-96T, Wuhan Servicebio Technology Co., Ltd., Wuhan, China), while glutathione (GSH) levels were measured with a reduced GSH assay kit (Cat. NO. G4305-48T, Wuhan Servicebio Technology Co., Ltd., Wuhan, China). All procedures were conducted in accordance with the manufacturer’s guidelines. The concentration of Fe^2+^ in the liver was quantified following the protocols outlined in the relevant kits (Cat. NO. 26641.96, Wuhan Servicebio Technology Co., Ltd., Wuhan, China).

### 4.10. Western Blot Assay

A BCA Protein Assay Kit (Thermo Fisher Scientific, Waltham, MA, USA) was used to quantify the total proteins that were isolated from liver tissues using a total protein extraction kit (Solarbio, Beijing, China). 12% SDS–PAGE was used to fractionate equal quantities of protein, which was then electrotransferred to PVDF membranes (240 mA, 90 min). The membranes were subsequently probed with primary antibodies for 8 h at 4 °C (Table S3). The secondary antibody employed was HRP-conjugated Affinipure Goat Anti-Rabbit IgG (H+L) from MedChemExpress, Shanghai, China, diluted to 1:5000. The membranes underwent TBST washes for 7 min, repeated five times, and were subsequently detected using the ECL Prime Western Blotting Detection Reagent (Shanghai Epizyme Biomedical Technology Co., Ltd., Shanghai, China). Band intensities were evaluated utilizing Image J software (Bio-Rad Laboratories, Berkeley, CA, USA).

### 4.11. Statistical Analysis

The data were examined with GraphPad Prism statistical software. All results are shown as mean ± standard deviation (SD). Substantial differences were assessed using one-way ANOVA with subsequent Bonferroni correction via GraphPad Prism 7. A *p*-value of <0.05 was deemed statistically significant.

## 5. Conclusions

Our studies illustrate the therapeutic efficacy of YDPG in liver fibrosis. This study utilizes a synthesis of network pharmacology, proteomics, and deep learning to investigate, for the first time, the mechanism of YDPG in the treatment of liver fibrosis from a comprehensive viewpoint. It offers more efficacious alternatives for the treatment of liver injury and associated disorders, as well as the exploration of multi-target pharmaceuticals.

While our data support a regulatory role of YDPG in the PPARγ/GPX4 axis, we acknowledge that direct evidence linking this pathway to ferroptosis inhibition remains incomplete. Future studies employing GPX4-knockout models or PPARγ antagonists are warranted to establish causal relationships. Nonetheless, the consistent upregulation of GPX4 and downregulation of Fe^2+^/MDA following YDPG treatment strongly suggest that this axis contributes to the anti-fibrotic effects observed.

## Data Availability

The original contributions presented in this study are included in the article/Supplementary Material. Further inquiries can be directed to the corresponding authors.

## Supplementary Materials

The following supporting information can be downloaded at https://www.mdpi.com/article/10.3390/ph19020251/s1. Table S1: Ishak scoring system for liver fibrosis; Table S2: Ishak scoring system for liver inflammation; Table S3: The antibody for western blotting; Table S4: Components identification results of YDPG.

## Abbreviations

YDPG	Yin Dan Ping Gan
HCC	Hepatocellular carcinoma
ALT	Alanine aminotransferase
AST	Aspartate transaminase
DBIL	Direct bilirubin
TBIL	Total bilirubin
γ-GT	Gamma-glutamyl transferase
TBA	Total bile acid
CCl_4_	Carbon tetrachloride
GSH	Glutathione
MDA	Malondialdehyde
HO-1	Heme oxygenase 1
H&E	Hematoxylin and eosin
SLC7A11, LC3A2	Solute carrier family 3 member 2; Solute carrier family 7 member 11
GPX4	Glutathione peroxidase 4
TFR	Transferrin receptor
NRF2	Nuclear factor erythroid 2
PPAR γ	Peroxisome proliferator-activated receptor γ
DTI	Drug-target interaction
FASP	Filter-aided sample preparation
GO	Gene ontology
BP	Biological processes
CC	Cellular components
KEGG	Kyoto Encyclopedia of Genes and Genomes
PPI	Protein–protein interaction
TCM	Traditional Chinese Medicine
