# Supplementary material for: Yin-Dan-Ping-Gan Capsule Mitigates CCL4-Induced Liver Fibrosis via Regulating PPAR γ/GPX4 Signaling and Suppressing Ferroptosis
Source: Pharmaceuticals (Basel). 2026 Feb 1;19(2):251. doi: 10.3390/ph19020251 (PMC12944426; doi:10.3390/ph19020251)
Supplement: Supplementary file 1 [file pharmaceuticals-19-00251-s001.zip › Table S1-3.pdf]

Table S1 Ishak scoring system for liver fibrosis

Lesion degree	Score
No fiber	0
Some portal areas have fibrous hyperplasia, with or without short fibrous septum	1
Most portal areas have fibrous hyperplasia, with or without short fibrous septum	2
Most of the portal areas have fibrous hyperplasia, and occasionally the portal areas are bridged by fibers	3
Fibrous hyperplasia in portal duct area with obvious fibrous bridging (between portal duct and portal duct and between portal duct and central vein)	4
Obvious bridging (portal tube to portal tube and /or portal tube to central vein) and occasional nodules (incomplete sclerosis)	5
Possible or definite cirrhosis	6

Table S2 Ishak scoring system for liver inflammation

Type	Lesion degree	Score
Focal (macular) lytic necrosis, apoptosis and focal inflammation	None	0
	There is 1 necrotic area or less per 10× visual field	1
	There are 2-4 necrotic areas per 10× visual field	2
	There are 5-10 necrotic areas per 10× visual field	3
	There are 10 necrotic areas per 10× visual field	4
Portal inflammation	None	0
	Mild, partial or all portal areas	1
	Moderate, partial or all portal areas	2
	Possible or definite cirrhosis	3
	Moderate / severe, all portal areas	4
	Serious, all gate tube areas	5

Table S3 The antibody for western blotting

Antibody	CAT	Brand	Dilution
Slc3a2	HY-P82453	MedChemExpress	1:1000
Slc7a11	HY-P80935	MedChemExpress	1:1000
Nrf2	HY-P81051	MedChemExpress	1:1000
PPAR-γ	HY-P80436	MedChemExpress	1:1000
CD36	HY-P81793	MedChemExpress	1:1000
GPX4	HY-P80450	MedChemExpress	1:1000
Keap1	HY-P80732	MedChemExpress	1:1000
GADPH	HY-P2804	MedChemExpress	1:5000
HO-1	YP-Ab-04799	UpingBio technology	1:1000
